# Detection and occurrence of microplastics in the stomach of commercial fish species from a municipal water supply lake in southwestern Nigeria

**DOI:** 10.1007/s11356-020-09031-5

**Published:** 2020-05-12

**Authors:** Aina O. Adeogun, Oju R. Ibor, Essa A. Khan, Azubuike V. Chukwuka, Emmanuel D. Omogbemi, Augustine Arukwe

**Affiliations:** 1 Department of Zoology, University of Ibadan, Ibadan, Nigeria; 2 Department of Biology, Norwegian University of Science and Technology (NTNU), Høgskoleringen 5, N-7491 Trondheim, Norway; 3 Department of Zoology and Environmental Biology, University of Calabar, Calabar, Nigeria; 4 National Environmental Standards and Regulation Enforcement Agency, Osogbo, Nigeria

**Keywords:** Microplastics, Feeding mode, Trophic level, Eleyele Lake, Tropical ecosystems

## Abstract

Microplastics (MPs) are physical anthropogenic pollutants and their ability to act as contaminant vectors in biological matrices is of serious ecosystem and human health concern. In the present study, we have, for the first time, screened and detected MPs in the stomach of a select group of commonly consumed fish species from a municipal water supply lake (Eleyele) in Nigeria. A total of 109 fish samples consisting of eight (8) species: *Coptodon zillii* (CZ: *n* = 38), *Oreochromis niloticus* (ON: *n* = 43), *Sarotheron melanotheron* (SM: *n* = 19), *Chrysicthys nigrodigitatus* (CN: *n* = 3), *Lates niloticus* (LN: *n* = 3), *Paranchanna obscura* (PO: *n* = 1), *Hemichromis fasiatus* (HF: *n* = 1), and *Hepsetus odoe* (HO: *n* = 1) were collected between February–April, 2018. Fish stomach content was screened for the presence of MPs using the density gradient separation technique (NaCl hypersaline solution) and examined using a fluorescence microscope. MPs were present in all the species screened (except *H. fasciatus*) with a frequency of 69.7% positive individuals in the examined species. MP prevalence was highest in ON (34%) > CZ (32%) > SM *(*13%) > CN (6%) and 5% each, for PO HO, and LN. On average, 1–6 MPs with sizes ranging between 124 μm and 1.53 mm were detected per individual. However, the highest number (34) of MPs was detected in the stomach of *SM*. Principal coordinate analysis (PCA) identified ecological variables such as habitat, feeding mode, and trophic levels as critical factors that may determine and influence MP uptake in fish population. The PCA showed stronger association between fish habitat, feeding mode, and trophic level with MP size and number in the benthopelagic species (ON CZ and SM), compared to demersal species (PO CN HO and LN). Given that MPs can act as vectors for the transfer of pathogens and environmental contaminants (both legacy and emerging), in addition to direct health risks to aquatic organisms, our findings raise concerns on the potential human/wildlife health effects of MPs in these economically and ecologically important food fishes.

## Introduction

The proliferation of plastics, particularly of polyethylene bags, polyethylene terephthalate (PET) bottles, and other single-use utility materials, has resulted to the annual introduction of approximately 300 million tons of plastic products into the environment (Nerland et al. 2014). Some authors have reported that an estimated five trillion plastics are floating particles on the ocean surface (Eriksen et al. 2014; PlasticsEurope 2016, 2017). The increased incidence of plastics in various compartments of the aquatic environment has been associated with human population density, highlighting a direct relationship between human population increase and plastic pollution (Engler 2012; Kurtela and Antolović 2019). Reports on the origin of microparticles (MPs) indicate that large plastic debris disintegrate and become smaller (< 1 mm) MPs via photolytic, mechanical, and biological degradation processes in the environment (Browne et al. 2007; Andrady 2011). These smaller particles are more bioavailable (Gregory 2009), with an increased surface area and greater likelihood of absorbing and desorbing toxic chemicals (Lee et al. 2014). Xenobiotic pollutants (including persistent organic pollutants—POPs) that can be absorbed upon ingestion by organisms provide routes for secondary toxicity and have been associated with MPs (Endo et al. 2005; Ziccardi et al. 2016; Koelmans et al. 2016). Other reported harmful consequences of MPs ingestion include endocrine, reproductive and developmental disruption, cellular and immune system damage, and negative impact on energy budget (Cole et al. 2015; Ogonowski et al. 2016; Pittura et al. 2018). The increased likelihood of aquatic organism to swallow and ingest MPs could also be of ecological concerns because they can be easily mistaken for food material, leading to artificial starvation and increased incidences of mortality. The indirect effects of MPs may include physical irritation, blockage of gills and occlusion of the digestive tract by smaller particles that are not easily dislodged (Laist 1997; Lambert and Wagner 2018).

Until recently, most studies in MPs pollution monitoring have focused on the marine ecosystem (Lambert and Wagner 2018). However, 80% of plastic waste originate from littering on land (Andrady 2011), making plastics and its degradation products major components of freshwater pollution (Williams and Simmons 1996; Balas et al. 2001; Eerkes-Medrano et al. 2015) and as vectors for the transfer of other contaminants into the aquatic environment (Koelmans et al. 2016). Concerns related to the incidence of these emerging pollutants in the aquatic environment have been largely attributed to their persistence, ubiquity, and toxic potential (Endo et al. 2005; Engler 2012; Kurtela and Antolović 2019). Further, once ingested, MPs may transfer through the habitat, feeding mode, and trophic level (Batel et al. 2016; Mattsson et al. 2015, 2017; Tosetto et al. 2017; Au et al. 2017; Windsor et al. 2019). These factors represent important variables that can influence MP uptake in biota from the surrounding environment (Desforges et al. 2015; Scherer et al. 2017; Bour et al. 2018b). While ecological factors such as habitat, feeding mode, and trophic level on MP uptake in biota have been suggested to play important roles (Au et al. 2017), very limited information is available on the influence of these ecological factors on MP uptake in aquatic organisms (Courtene-Jones et al. 2017; Lusher et al. 2013; Bour et al. 2018b). In particular, such information is non-existent from developing countries, including Nigeria. Therefore, understanding the influence of ecological and biological factors on the distribution of MPs across food webs is urgently needed as an initial step towards monitoring and mitigation of plastic pollution.

Issues regarding species-specific risks and the extent to which organisms are likely to take-up MPs in the environment still remains an open ecological question about plastic pollution that is yet to be addressed (Scherer et al. 2018). This becomes necessary because MPs are important component of aquatic ecosystems and particle properties such as size distribution, shape, concentration and chemical composition, and duration of exposure play strong roles in determining their interactions with aquatic species and communities (Scherer et al. 2018). While several lentic and lotic ecosystems have been investigated for MP occurrence and distributions in many developed countries, the dynamics of MP distribution, fate, transfer routes, habitat influence, feeding mode, and trophic level in biota within urban-catchments and hydro-systems of tropical environments are generally unknown.

The West African aquatic ecosystems are considered biodiversity hotspots for several aquatic fauna. However, the narrow study on MPs contamination in this region is a singular report on the incidence of MPs in *Lates niloticus* and *Oreochromis niloticus* from the Mwana region of Tanzania, south of Lake Victoria (Biginagwa et al. 2016). This suggests an urgent need to understand the relationships between MP occurrence in biota and population level impacts. Such studies will include the influence of habitat, trophic level, and feeding mode which are considered necessary in determining the bioavailability of MPs and the broader implications for ecosystem functioning. Therefore, the present study was designed with the aim of detecting the occurrence and distribution of MPs in the stomach of some commonly consumed fish species from a municipal water supple lake (Eleyele). Specifically, the study addressed the following specific questions: (1) relative prevalence and abundance of MPs in the stomach of fish species from Eleyele Lake, (2) size distribution of MPs across species, and (3) the influence of habitat, trophic level, and feeding mode on MP distribution and uptake across these fish species.

## Materials and methods

### Chemicals and reagents

Sodium chloride (NaCl) and hydrogen peroxide (H_2_O_2_) were purchased from Sigma-Aldrich Oslo, Norway. Other reagents and chemicals used in the study were of the highest commercially available analytical grades.

### Study area

**Fig. 1:**
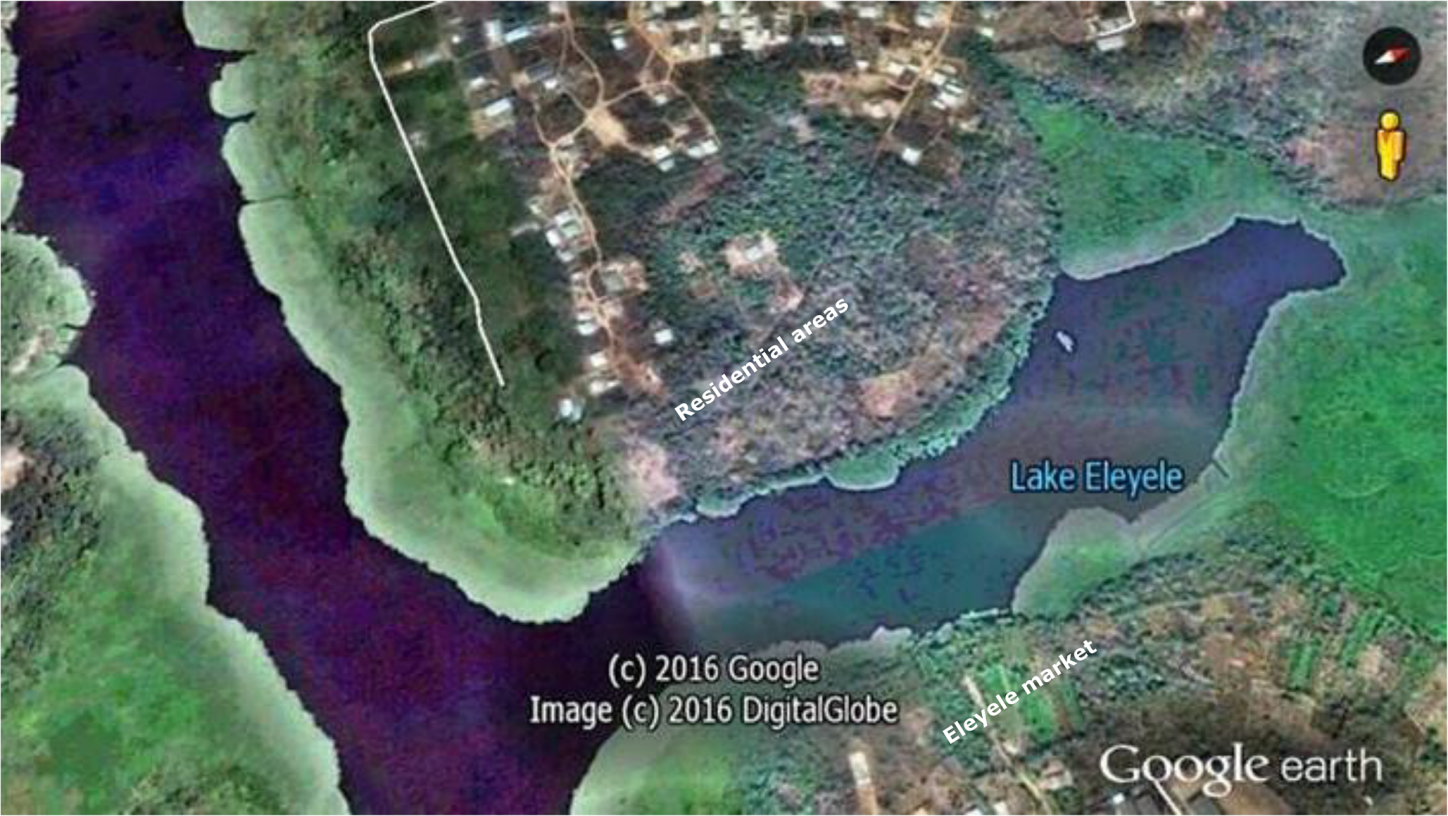
Map of Eleyele, a municipal water supply lake in southwestern, Nigeria

Eleyele Lake is a municipal water supply lake that supplies water to the Ibadan municipality and is located within the Eleyele catchment area in Ido local Government Area, Ibadan, Oyo State Southwest-Nigeria (Fig. 1). The lake was formed through a damming process of River Ona River, the streams of Awba, Otaru, Yemoja, and Alapo. It is located at an altitude of 125 m above sea level and lies within Latitude 7°25′–7°26′N and Longitude 3°51′–3°52′E (Fig. 1). The lake covers a distance of 62 km source to the dam site, is flood controlled, and has a total impoundment area of 5.46 km^2^ and a maximum depth of 12 m during the flood season. At the time of creating the dam, the initial water storage capacity was 704 million liters (Imevbore 1967), and this was later increased to 900 million liters to meet the increasing demand for domestic water supply to the Ibadan municipality. Plastic items of different sizes and shapes (plastic bottles and polythene bags of different polymer types) used mainly in household, personal care products, and construction were observed floating on the surface of the lake with higher aggregation close to the landward portions of the lake.

### Sample collection

**Fig. 2:**
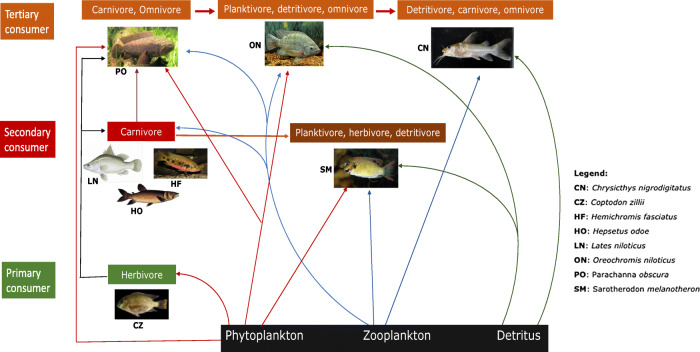
Habitat preference and trophic web location of the studied fish species

A total of 109 fish samples consisting of eight (8) species: *Coptodon zillii* (CZ: *n* = 38), *Oreochromis niloticus* (ON: *n* = 43), *Sarotheron melanotheron* (SM: *n* = 19), *Chrysicthys nigrodigitatus* (CN: *n* = 3), *Lates niloticus* (LN: *n* = 3), *Paranchanna obscura* (PO: *n* = 1), *Hemichromis fasiatus* (HF: *n* = 1), and *Hepsetus odoe* (HO: *n* = 1) samples were collected randomly from the entire length of Eleyele Lake between February–April, 2018 with the aid of artisanal fishermen. All fish species were caught using cast net with mesh sizes of 50–55 mm. Fish were anesthetized on ice and transported to the laboratory. Fish morphometric data, including body weight (BW) and gut weight (GW), were measured with an Ohaun digital weighing balance (Mettler Instruments), while total length (TL) and standard length (SL) were measured with an absolute digital caliper (Tresna Instruments). Fish condition factor (k) was calculated as condition factor (k) = 100 × W/L^3^, where W = fish body weight and L = total length (Fulton 1902). Fish sampling was planned to target fish species at different trophic levels within the Eleyele lake catchment (Fig. 2).

### MP extraction, detection and quantification

Prior to dissecting fish for MP extraction, detection, and quantification, the entire fish exterior was rinsed with Milli-Q water, before stomach dissection for examination using the procedures reported by Avio et al. (2015) and Bour et al. (2018), with slight modifications. The density gradient separation method was used for detecting MPs in stomach contents. Briefly, fish stomach was opened by horizontal slicing of the stomach and thereafter emptied into a 500-mL glass beaker and 250 mL of NaCl hypersaline solution (1.2 g/cm^3^) prepared using Milli-Q water. The sample solution was stirred for 10 min, allowed to settle for another 10 min, and thereafter, the supernatant was decanted into a new 500-mL glass beaker and the extraction step was repeated again. The supernatant was filtered under vacuum onto a PVDF membrane (0.45 μm), and filtration was carried out twice in order to obtain a better extraction performance and higher MP recovery. After filtration, the membrane was immediately transferred into a glass petri dish, digested with 2 mL of 15% H_2_O_2_, and allowed to dry in the oven at 50 °C overnight. The membranes were thereafter examined under the fluorescence stereo zoom microscope (Ziess Axio Zoom V16). MPs were then photographed with size measurement, as the largest cross-section through the ocular micrometer and the number of fluorescence MP particles, which were counted for each membrane and recorded.

### Quality assurance

To avoid potential MP contamination of all our experimental samples, reagents, equipments, and clothing, we performed a laboratory control experiment with all listed materials in our procedures. The same nitrile gloves and laboratory coat materials were worn during the field sampling to replicate similar experimental conditions during sampling, and all laboratory analysis including fish stomach dissection and MPs analysis were performed inside a fume hood. All glass wares, filters, dissecting sets, and tiring apparatus were washed and rinsed with absolute ethanol, autoclaved, and properly covered with aluminum foil and placed in the fume hood. A quality control assurance test was also conducted in order to ascertain absence of contaminant particles in reagents and equipment including NaCl, H_2_O_2_, Milli-Q water, PVDF membrane, filters, glass petri dishes, and glass wares to ensure that they do not contain particles that can cause contamination. All experimental procedures were similar for both the quality assurance test and sample analysis as stated above. No visible MPs particle fluorescence was observed on the PVDF membrane (see Fig. 4a) indicating absence of contaminants and the suitability of the PVDF membrane for the filtration of MPs.

### Statistical analysis

Data obtained were presented as mean ± SEM while percentages and frequency of microplastic occurrence in species and entire sampled population were also performed using Prism GraphPad 5 (GraphPad software, La Jolla, USA). Principal coordinate analysis (PCoA) was performed to determine the influence of ecological variables (habitat, feeding mode, and trophic level) on MP uptake and size distribution across species using SPSS version 21.

## Results

### Occurrence, distribution, and abundance of MP across species

**Fig. 3:**
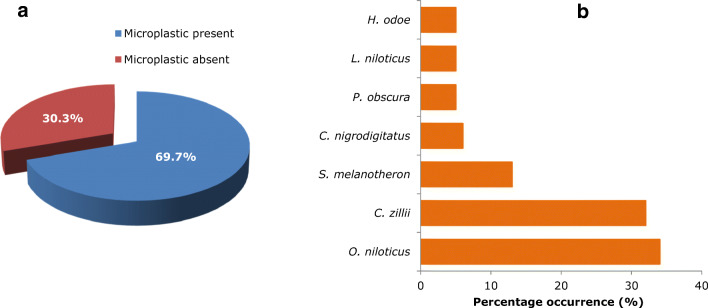
Occurrence and distribution of microplastics in some commonly consumed fish species from Eleyele Lake. **a** Overall prevalence of microplastics in commercial species. **b** Occurrence of microplastics in different commercial fish species

A total of 109 fish belonging to eight (8) species and difference habitat, trophic level, and feeding mode were analyzed showing a 69.7% prevalence of MPs, while only 30.3% of the sampled fish population had stomachs without MPs (Fig. 3a). MPs were detected in seven (7) out of the eight (8) species analyzed, except *Hemichromis fasciatus* (HF). The highest MP occurrence was recorded in *O. niloticus* (ON) with 34%, followed by *C. zillii* (CZ) with 32% and *S. melanotheron* (SM) with 13%, while the least was recorded in *P. obscura* (PO), *L. niloticus* (LN), and *H. odoe* (HO) which accounted for 5% each of MP occurrence in the sampled population (Fig. 3b). Also, MP prevalence across species was in the order *ON* > *CZ* > *SM* > *CN* = *PO = LN = HO* (Fig. 3b). It is important to note that the occurrence and prevalence of MPs directly reflects the number of fish (i.e., *n*) samples for each species.

### Morphometric measurements, MP number, and sizes across species

The morphometric measurements including the number and size of MPs across species are presented in Table 1, showing that CN had the highest total length (32.0 ± 1.8 cm), standard length (28.0 ± 2.0 cm), and bodyweight (337.5 ± 1.5 g), while LN had the best condition factor (k) of 3 ± 1. *PO* had the highest gutted weight (4.1 g), compared with all other species examined (Table 1).

**Table 1:** Fish biometric data, habitat, number, and sizes of microplastics (MPs) recorded

	Habitat	TL	SL	BW	K	GW	No. of MPs	Size of MPs
*Coptodon zillii* (*n* = 38*)*	Pelagic	17.6 ± 0.4	13.8 ± 0.3	109.5 ± 7.0	1.9 ± 0.1	1.8 ± 0.2	1–6	126 μm −1.5 mm
*Oreochromis niloticus* (*n* = 43)	Benthopelagic	16.5 ± 0.3	12.9 ± 0.2	96.0 ± 4.7	2.1 ± 0.1	1.8 ± 0.1	1–4	124 μm −1.3 mm
*Sarotherodon melanotheron* (*n* = 19)	Benthopelagic	17.0 ± 0.4	13.2 ± 0.4	99.6 ± 8.6	2.0 ± 0.3	2.0 ± 0.1	1–34	1–1.02 μm
*Chrysicthys nigrodigitatus* (*n* = 3)	Benthic/Demersal	32.0 ± 1.8	28.0 ± 2.0	337.5 ± 1.5	1.2 ± 0.3	0.9 ± 0.1	1–3	1–1.28 μm
*Lates niloticus* (*n* = 3)	Pelagic	21.0 ± 0.4	9.30 ± 2.9	97.5 ± 3.5	3.0 ± 1.0	1.1 ± 0.2	1–4	1–1.15 μm
*Parachanna obscura* (*n* = 1)	Benthic/Demersal	23.3	18.5	84	0.7	4.1	1–5	1–1.24 μm
*Hepsetus odoe* (*n* = 1*)*	Pelagic	21.4	14.4	84.0	0.9	4.0	1–2	1–1.53 μm
*Hemichromis fasciatus* (*n* = 1)	Pelagic	17.9	14.2	128.0	2.0	2.23	–	–

*TL* total length, *SL* standard length, *BW* body weight, *K* condition factor, *GW* gutted weight, *No. MPs* = number of microplastics, *Size of MPs* size of microplastics

**Fig. 4:**
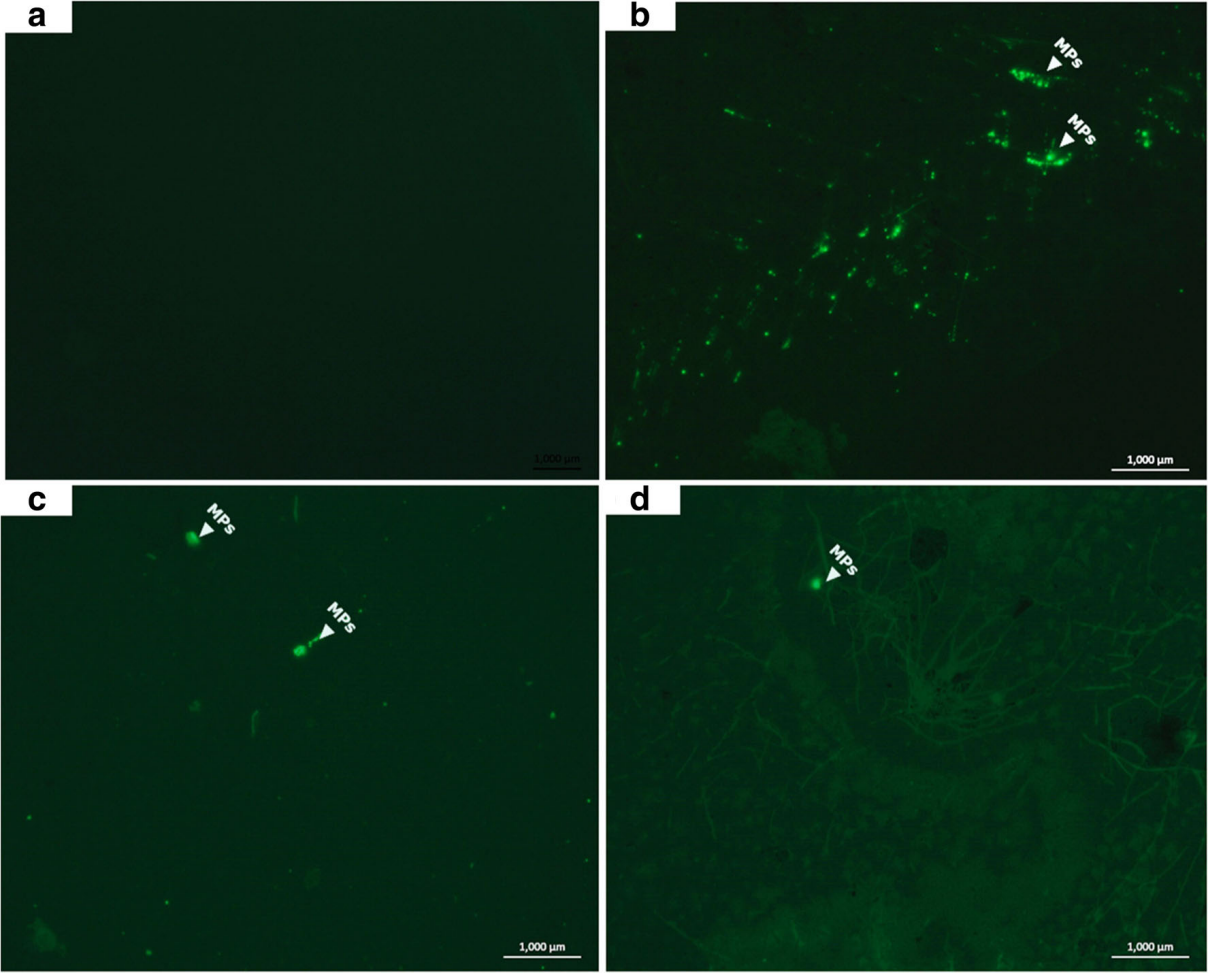
Fluorescence photomicrographs showing microscopic detection of microplastics in the stomach of food fish species from Eleyele Lake. **a** Control (blank PVDF membrane). **b***Sarotheodon melanotheron*. **c***Coptodon zillii*. **d***Oreochromis niloticus*

**Fig. 5:**
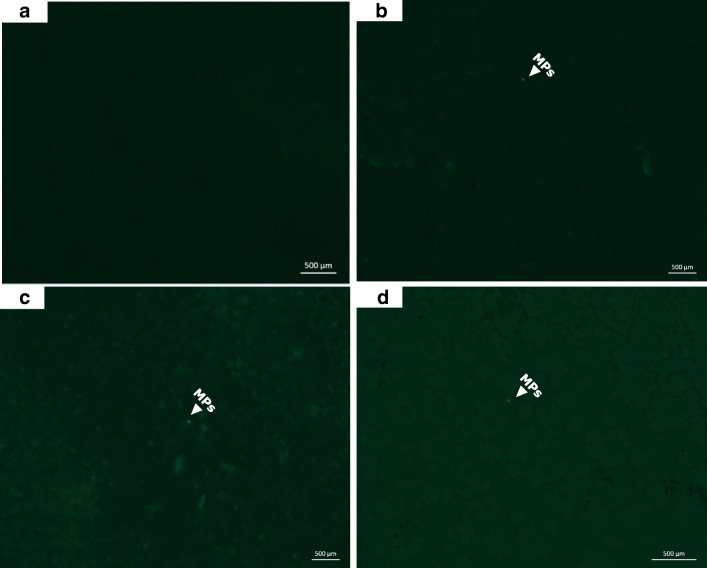
Detection of microplastics in the stomachs of food fish species. **a***Hermichromis fasciatus*. **b***Chrysicthys nigrodigitatus*. **c***H. odoe*. **d***Parachanna obscura*

The highest number of MPs in fish stomach was recorded in the benthopelagic species (*S. melanotheron* and *C. zillii*) with a range of 1–34 and 1–6 MPs, respectively. These species are primary consumers with herbivorous, detrivorous, and planktivorous mode of feeding, respectively. The least MPs occurrence was recorded in *H. odoe*, a carnivorous, pelagic species and a secondary consumer (Table 1). The smallest MP sizes ranging between 124 and 126 μm were only recorded in *O. niloticus* and *C. zillii* (Table 1). Fluorescence images of MPs particles detected in fish stomachs across sampled fish species are presented in Figs. 4 and 5, respectively. Specifically, no evidence of fluorescence MPs was observed in the blank PVDF membrane used as control to validate the absence of MPs on the membranes used (Fig. 4a), while fluorescence MPs particles were observed in *S. melanotheron*, *C. zillii*, and *O. niloticus* (Fig. 4b–d). Similarly, there was no fluorescence MP in the analyzed stomach of *H. fasciatus* (Fig. 5a), while few fluorescence MPs particles were observed in *C. nigridigitatus*, *H. odoe*, and *P. obscura* (Fig. 5b–d, respectively).

### Principal coordinate analysis

**Fig. 6:**
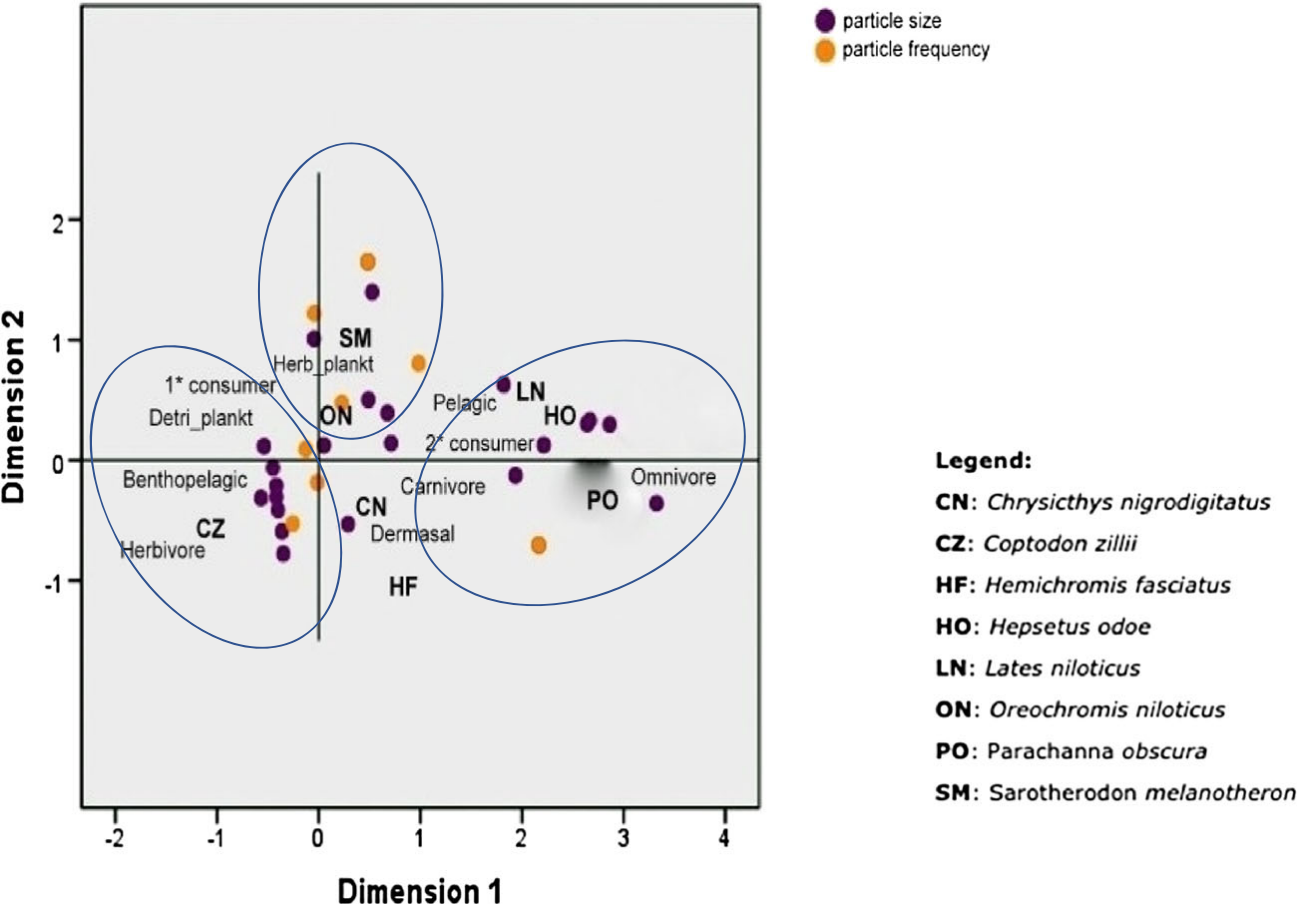
Principal coordinate analysis (PCoA), showing the relationship between ecological habitat, feeding mode, and trophic level with MP uptake and sizes in different food fish species from Eleyele Lake

Principal coordinate analysis (PCoA) of the correlations between particle size and distribution, fish feeding mode, habitat, and trophic level is shown in Fig. 6. PCoA 1 showed very strong positive association between particle size recovered from the stomach of *C. zillii*, mainly herbivorous feeding mode, pelagic habitat, and trophic level (i.e., primary consumer), revealing that feeding on plankton/water plants (MPs may adhere to water plants) had strong influence on the wide range in size of MPs (126 μm–1.5 mm) recovered from the stomach of *C. zillii*. The weak association between these variables for *P. obscura* (an omnivore with carnivorous tendencies: tertiary consumer) on the other hand is an indication that feeding mode and trophic level are important variables for MP particle size in fish species from Eleyele Lake. Particle size correlation was in the order *CZ* > *PO* > *CN*. Strong positive associations were also observed for *O. niloticus* on PCoA 2 axis with the association trend in the order *ON* > *SM* > *HO* > *LN*. The correlation between particle frequency and other variables on axis 1 showed moderate positive correlations with CZ and was in the order of CZ > PO > CN. A strong positive association was observed for particle frequency in *S. melanotheron* indicating that the ability of SM to explore a wide variety of feeding modes (herbivore, planktivore and detritivore) and trophic level (primary consumer) was important variables in the high number of MPs recovered from stomach and also evident with the high abundance (*n* = 19) of this species in the sample material. The trend of association was in the order SM > CZ = ON. Such associations were not observed for LN and HO (carnivores). No association was found between all variables for *Hemichromis fasciatus* which may indicate the absence of MPs in the stomach of this species from Eleyele Lake. However, the number of HF in the sample population was very low (*n* = 1) and may account for the no association trend observed in the plots.

## Discussion

Environmental monitoring of MPs occurrence in aquatic ecosystems has received increased attention in recent times because of reports identifying MPs as vectors that may enhance the transfer of several pathogens and contaminants of emerging concern (including polycyclic aromatic hydrocarbons, bisphenol-A and polychlorinated biphenyls) in aquatic environment and biota (Williams and Simmons 1996; Balas et al. 2001; Endo et al. 2005; Carson et al. 2013; Eerkes-Medrano et al. 2015; Batel et al. 2016; Koelmans et al. 2016; Viršek et al. 2017; Pittura et al. 2018). Several reports have focused on identification and characterization of MPs in different aquatic environment and biota matrices in order to determine the extent and severity of plastic pollution. These legitimate concerns and the toxic impacts of MPs for aquatic organisms and human health is supported by the fact that adsorbed pollutants may leach out upon ingestion, providing routes for secondary toxicity due to bioaccumulation and biomagnification along the food web (Ziccardi et al. 2016; Endo and Koelmans 2016; Koelmans et al. 2016). While plastic pollution has received global attention with urgent and priority response from government, researchers, and ecotoxicologist in many developed countries, information on occurrence of MPs in commercial fish species is limited or non-existent in Africa, except a singular report on *Lates niloticus* and *Oreochromis niloticus* from the Mwana region of Tanzania located on the south of Lake Victoria (Biginagwa et al. 2016). We demonstrated, for the first time, in any aquatic and terrestrial species or lower vertebrate, the occurrence and prevalence of MPs in the stomach of some commonly consumed freshwater fish species from a municipal water supply lake, in southwest, Nigeria. We showed a 69.7% prevalence of MPs in about 109 sampled fish and observed the presence of MPs in seven (7) out of the eight (8) species, indicating the widespread MP contamination in these species. Further, multivariate correlation using principal coordinates analysis (PCoA) highlighted ecological variables such as habitat, feeding mode, and trophic levels as critical factors that determine and influence MP uptake in fish population from the Eleyele Lake.

The MP prevalence of 69.7% in this study may be a reflection of the extent, magnitude, and abundance of plastic contamination at the Eleyele Lake ecosystem and may explain the high rate of MP ingestion by resident biota. These findings are comparable with previous reports on MP prevalence and ingestion in the gastrointestinal tracts of Japanese anchovies from the Tokyo Bay (Tanaka and Takada 2016); fish from the Costa Concordia wreck, at Giglio Island (Avio et al. 2017), Baltic sea (Gewert et al. 2017); fish from Jeløya, Norway (Bour et al. 2018b); and sardines from the Northern Ionian sea (Digka et al. 2018). Unlike the observed high MP prevalence observed in our study, lower MPs prevalence has been reported elsewhere in other fish studies. For example, Boerger et al. (2010) reported 35% MPs prevalence in planktivorous fishes from the North Pacific Gyre of California, 33% prevalence in fish from the Goiana estuary in Brazil (Possatto et al. 2011), while 18.2% prevalence was reported from the Mediterranean Sea, Messina Italy (Romeo et al. 2015). We believe that the differences observed in MPs prevalence between these reports and our study may be related to the different features and characteristics of the environments and preference in the trophic web. Most of these reports have used marine and brackish water fish species with characteristic high-water volume, flow rate, wave action, and serial dilution of materials, while our study was on a freshwater lentic lake characterized by low dilution and flow rate, highlighting the possibility of the observed MPs prevalence differences. Furthermore, our observed high MPs in fish stomach, compared with the above-mentioned reports, may be related to the anthropogenic pressure faced by the different ecosystems; for example, large surrounding land-areas characteristic of lacustrine ecosystems may increase the possibility of anthropogenic inputs from adjacent terrestrial environments, compared with oceans and estuaries. This argument is supported by the fact that 80% of plastic wastes into the aquatic environment originate from littering on land (Andrady 2011). This could also explain the higher MPs prevalence observed in the present study.

The highest number of MPs recovered from the stomachs of the benthopelagic species (*S. melanotheron*) and pelagic species (*C. zillii*) is probably due to the fact that both species have overlapping feeding modes (herbivores, plantktivores and detrivore) and occupy the same trophic level (primary consumers), indicating that habitat, feeding mode, and trophic level are ecological factors that could determine the incidence and abundance of MPs ingestion in fish. We believe that the higher MPs in these benthopelagic species may be indicative of their ability to interact more with MPs adsorbed to particulate matter within sediment. On the other hand and regardless of the low number of samples, the pelagic species and carnivore (*H. odoe*) had the least number of MPs (1–2) in their stomach and this may be related to their habitat preference and feeding habit as piscivores (feed on other fish species), with lower likelihood to interact with MPs either through filter feeding or sediment particles. The number of MPs recovered from fish stomach in this study are comparable with other previous reports of MP contamination from the coastal waters of China (Qiu et al. 2015; Li et al. 2016) and UK (Li et al. 2018); estuaries of Portugal, Italy, and Spain (Vandermeersch et al. 2015); Northern Ionian Sea (Digka et al. 2018), Adriatic Sea (Avio et al. 2015); and Mediterranean coast of Spain (Bellas et al. 2016), Turkey (Güven et al. 2017), Belgium (De Witte et al. 2014), and Norway (Bour et al. 2018). We detected MPs sizes ranging from 124 μm to 1.5 mm in *C. zillii*, *O. niloticus*, and *S. melanotheron* while other species had MPs sizes ranging between 1 and 1.53 mm. These indicate that there are smaller MPs in the benthopelagic, compared with the pelagic and dermersal species and in the primary consumers than in other higher trophic level species. Our ability to recover MPs particle size of about 124 μm may be related to the nature of the MPs present in these species and suitability of our applied analytical method (the use of membrane of 0.45 μm pore size) to recover more and smaller particles. Previously, analytical detection methods and sample processing have been highlighted as critical factors that determine MP size recovery (Bour et al. 2018). In accordance with our findings, MP sizes ranging from 0.41 μm to 9 mm were reported in fish from the Jeløya, Norway (Bour et al. 2018), 0.5 - 1 mm, 0.1–0.5 mm, 1–5 mm in fish from the Giglio Island (Avio et al. 2017), 0.1–1 mm in fish from the Adriatic Sea (Avio et al. 2015), 41 and > 1 mm pelagic MPs from Michigan Lake (Mason et al. 2016).

The highest species occurrence of MPs in our study was recorded in the benthopelagic species in the order *O. niloticus* > *C. zillii* > *S. melanotheron* with a prevalence of 34, 32, and 13% respectively. Interestingly, correlational analysis by principal coordinates analysis (PCoA) identified ecological variables such as habitat, feeding mode, and trophic levels as critical factors that determine and influence MP uptake in the fish population from Eleyele Lake. This indicates that particle size and frequency, ecology (benthopelagic and pelagic habitats), and biological variables such as trophic level and feeding mode (herbivorous and planktivorous/detritivorous/ feeding habits) were major determinants in MP distribution in *S. melanotheron*, *C. zillii*, and *O. niloticus*, respectively. The pelagic carnivores and demersal omnivores belonging to the secondary and tertiary consumers trophic levels (*H. odoe*, *L. niloticu*s, and *C. nigrodigitatus*) showed the leaser association with MP prevalence, particle sizes, and frequency. These relationships may suggest that ecological variables such as habitat, feeding mode, and trophic level are important variables that may influence the rate of MP uptake and subsequent prevalence in organisms. The observed higher association between MPs uptake, sizes, feeding mode, and trophic level in the benthopelagic and pelagic species may be related to their ability to explore a wider ecological habitat for different feed sources and their filter feeding mode which may allow them to ingest more MPs in the water column, compared with the pelagic/demersal species that are secondary consumers and are restricted to a narrower ecological niche for food. This assumption is supported by established knowledge indicating that higher MPs concentrations in filter feeders, compared with the other species (Karlsson et al. 2017; Leslie et al. 2017).

The presence of a smooth branchial apparatus in the filter feeders may also enhance their ability to filter and take up smaller MPs from the water column with high efficiency (Collard et al. 2017). Thus, explaining the observed high association and prevalence observed in this species. Similar to our findings, higher MP occurrence has been reported in benthopelagic, compared with pelagic/demersal species (Bessa et al. 2018; Digka et al. 2018), and the influence of feeding type in MPs ingestion in aquatic species has been previously reported (Setälä et al. 2016; Mizraji et al. 2017). However, due to the limited number of pelagic/demersal species (*n* = 5 in total for LN, HO, and HF respectively) used in this study, we highlight the need for more studies on these species for MPs prevalence in field studies.

In conclusion, our findings provide the first evidence on the occurrence and prevalence of MPs in food species from a tropical and municipal water supply lake (Eleyele) from Nigeria. We have identified the extent and severity of MP contamination in food fish species and highlight the need for more studies to address wildlife and human health consequences of MP contamination in this important aquatic food species. Further, given the role(s) of MPs as direct toxic materials and vectors for the transfer of several pathogens and environmental contaminants of emerging concern in the aquatic environment, the information presented herein is an ongoing effort to provide important research materials that are targeted towards food, human health, wildlife, and environmental safety in Nigeria in particular and developing countries in general.
